# Heavy Draught Breeding Horses

**Published:** 1881-01

**Authors:** 


HEAVY DRAUGHT BREEDING HORSES seem to be in greater demand in the United States than even before the days of rail-roads. During 1876 some three hundred were imported from Europe, and the prospect now is that even that number will be exceeded this year. These heavy horses are not popular in the Eastern cities, however; it is found that the most work can be gotten out of medium-sized animals. In cities, especially, large horses have to be used with great care, as any rapid driving over the paved streets is sure to injure the hoofs. Heavy draught horses cannot be used in cities until they are well matured.

